# Perspectives on sexual and reproductive health self-care among women, healthcare providers, and other key informants: a mixed-methods study in South Africa and Zambia

**DOI:** 10.1186/s12978-023-01596-x

**Published:** 2023-04-28

**Authors:** Alice F. Cartwright, Marissa Velarde, Mags Beksinska, Jennifer Smit, Margaret Kasaro, Jennifer H. Tang, Cecilia Milford, Virginia Maphumulo, Manze Chinyama, Esther Chabu, Mayaba Mudenda, Christina Wong, Maria Fawzy, Rebecca Callahan

**Affiliations:** 1 FHI 360, 359 Blackwell St, Suite 200, Durham, NC 27701 USA; 2 MRU (MatCH Research Unit), Department of Obstetrics and Gynecology, Faculty of Health Sciences, University of the Witwatersrand, Durban, South Africa; 3 Division of Global Women’s Health, Department of Obstetrics and Gynecology, University of North Carolina (UNC) at Chapel Hill, Chapel Hill, NC USA; 4 UNC Global Projects, Lusaka, Zambia; 5 Department of Maternal and Child Health, Gillings School of Global Public Health, University of North Carolina at Chapel Hill, Chapel Hill, NC USA

**Keywords:** Self-care, Family planning, Contraception, Sub-Saharan Africa, IUD self-removal

## Abstract

**Background:**

“Self-care” for sexual and reproductive health (SRH) includes contraceptive methods and other supplies that people can use with or without the support of a healthcare provider. Self-administered tests, self-injection of injectable contraception, or self-removal of intrauterine devices (IUDs) can increase people’s access to and autonomy over their own SRH. Objectives of this study were to assess women’s current interest in and use of SRH self-care and explore key informants’ (KI) opinions of self-care, especially during the COVID-19 pandemic.

**Methods:**

Data for this study came from female participants in the longitudinal Contraceptive Use Beyond ECHO (CUBE) study, and KIs, including healthcare providers, in South Africa and Zambia between September 2020 and June 2021. For this analysis, we used data from a participant phone survey (n = 537), and from in-depth interviews (IDIs) completed with a sub-sample of women (n = 39) and KIs (n = 36). Survey data were analyzed with descriptive statistics, and IDI data were analyzed using applied thematic analysis.

**Results:**

Female survey participants in South Africa were more interested in learning about emergency contraceptive pills, subcutaneous injectable contraception, and CycleBeads, while Zambian participants wanted more information and access to condoms. However, in IDIs in both countries, women described minimal experience with self-care beyond condom use. In the Zambian KI IDIs, COVID-19 led to increased self-care counseling on subcutaneous injectable contraception and HIV self-testing. KIs who do not counsel on self-care were concerned that women may harm themselves or blame the provider for difficulties. Two KIs thought that women could possibly self-remove IUDs, but most expressed concerns. Reported barriers to self-care included COVID movement restrictions, transport costs, lack of accessible pharmacies, women’s low awareness, and possible stigma.

**Conclusions:**

Women surveyed reported interest in learning more about SRH self-care methods and resources, but in IDIs did not report extensive previous use besides condoms. KIs described some concerns about women’s ability to use self-care methods. Counseling on and provision of self-care methods and supplies may have increased during the COVID-19 pandemic, but ensuring that self-care is more than just a temporary measure in health systems has the potential to increase access to SRH care and support women’s autonomy and healthcare needs.

**Supplementary Information:**

The online version contains supplementary material available at 10.1186/s12978-023-01596-x.

## Plain Language Summary

**Background:**

“Self-care” refers to healthcare that does not have to be given by a provider, but that people can use themselves. In sexual and reproductive health (SRH), this includes medicines or supplies like pills and injections that people can use to prevent or test for pregnancy or sexually transmitted infections. This study wanted to better understand women’s interest in and use of SRH self-care and explore key informants’ opinions of self-care, especially during the COVID-19 pandemic.

**Methods:**

We surveyed 537 women in KwaZulu-Natal province, South Africa and Lusaka, Zambia in 2020–2021. We also conducted interviews with 39 women and 36 key informants, including healthcare providers, government officials, and community advocates.

**Results:**

Women surveyed in South Africa were more interested than those in Zambia in learning more about self-care contraception, especially daily pills, emergency pills, and injections they could give themselves. In interviews, some key informants said that they do not tell women about self-care because they worried that women could hurt themselves or blame the provider if they experienced problems. COVID movement restrictions, transport costs, and inaccessible pharmacies were all barriers that key informants mentioned to accessing tests, tools, or contraceptive methods that women could give or use themselves.

**Conclusions:**

Women surveyed were interested in learning more about self-care and those interviewed reported minimal previous use of self-care methods besides condoms. Providers also have some concerns about women’s ability to use self-care methods. Counseling on and providing self-care methods and supplies may have increased during COVID-19, but increasing access to self-care could help more women take care of their own sexual and reproductive healthcare.

**Supplementary Information:**

The online version contains supplementary material available at 10.1186/s12978-023-01596-x.

## Background

In guidelines released in 2019, the World Health Organization (WHO) defined self-care as “the ability of individuals, families and communities to promote health, prevent disease, maintain health, and cope with illness and disability with or without the support of a healthcare provider” [1]. This definition is broad and meant to comprise many health-promoting activities, including those related to sexual and reproductive health (SRH). Self-care interventions for SRH are diverse, encompassing self-screening or testing for sexually transmitted infections (STIs), HIV, or pregnancy, as well as contraception, such as condoms, over-the-counter oral contraceptives (OCs), emergency contraceptive pills (ECPs), or self-administered injectable contraception [1].

Some self-care interventions like condoms have been used for decades, while others, such as self-injectable contraception, are being rapidly introduced [2]. As has been the trend across many sub-Saharan African countries in recent years, Demographic and Health Survey (DHS) data from South Africa and Zambia indicate that the use of some self-care methods continues to increase [3, 4]. For example, in South Africa, male condom use for pregnancy prevention increased from 2% in 1998 to 16% in 2016 among sexually active women aged 15–49, and almost half of condom users obtained them from a pharmacy, shop, or other outlet, rather than a health facility [4]. While reported use of ECPs is still relatively low in these countries, about a third of women in Zambia [3] and 63% of women in South Africa have knowledge of ECPs [4], and 85% of women in a study in Johannesburg reported willingness to use ECPs [5].

In both countries, the most popular contraceptive method is injectable contraception (accounting for approximately half of the method mix), though most of these injections are administered by providers [3, 4]. Recent studies have shown that self-administered injectables are both feasible and acceptable and improve contraceptive continuation among users compared with provider administration [6–8]. Evidence from Zambia indicates that women can self-inject successfully and can perform additional injections on time without interactions with a provider [9]. Self-care has the potential to increase contraceptive privacy and autonomy, and access to methods, while also reducing dependence on health facilities and overburdened health workers during the COVID-19 pandemic and beyond [2, 10–13]. Similarly, self-testing for pregnancy, HIV, and other STIs has been well-studied, and research has shown that self-testing is an accurate and acceptable alternative to provider testing [14–17].

A newer area of contraceptive self-care is self-removal of intrauterine devices (IUDs), which allows people more autonomy over when they discontinue IUD use (without having to return to a provider for removal). Only one study in the United States (US) has actually tested success rates of IUD self-removal [18], though another US study indicated that people were more interested in using an IUD when provided information about the possibility of self-removal [19]. To date, no study has explored perceptions of IUD self-removal in low and middle income countries.

Access to and uptake of some self-care interventions are also sometimes facilitated by healthcare providers, who must be receptive and willing to integrate it into their practice. Healthcare providers may be resistant to these changes or feel challenged by new forms of self-administration of contraception, self-testing, or task-sharing of SRH services due to concerns for their clients’ safety as well as concerns about the job security in their roles as providers [1, 12, 20]. Except for a global online survey conducted in 2018 with respondents from 112 countries [20], research on healthcare providers’ views of the wide spectrum of self-care contraceptive and SRH methods is limited. Providers’ perspectives on self-administration of injectable contraception suggest that the majority find it acceptable and prefer it over provider-administered injectables [21, 22]. On the other hand, providers have conflicting opinions regarding the use of self-testing for HPV and HIV, with some expressing skepticism and others strong support [23, 24]. In the aforementioned global survey, providers noted sociocultural issues such as shame, stigma, or lack of supportive policies and accessibility as barriers to SRH self-care, but they also noted that self-care interventions may be helpful for young people and marginalized individuals, offering them confidentiality and ease of access [20].

The objective of this study was to assess women’s current interest in and use of SRH self-care, particularly as it relates to contraception, and explore key informant (including healthcare provider) opinions of self-care in South Africa and Zambia. This study includes primary analyses of secondary objectives from an additional survey module conducted during the COVID-19 pandemic. Therefore, this study specifically presented an opportunity to investigate how women and healthcare providers were adapting their SRH self-care practices amid the crisis.

## Methods

### Study design

This analysis uses data from a COVID-19 survey and qualitative in-depth interviews (IDIs) with contraceptive users, providers, and other stakeholders participating in the Contraceptive Use Beyond ECHO (CUBE) study. The CUBE methods have been described in detail previously [25, 26]. Briefly, a sample of participants who had recently completed their final study visit for the Evidence for Contraceptive Options and HIV Outcomes (ECHO) trial in KwaZulu-Natal province, South Africa and Lusaka, Zambia were recruited to participate in CUBE. Eligible participants had to be using one of the ECHO contraceptive methods at exit: intramuscular depot medroxyprogesterone acetate 3-monthly injectable (DMPA-IM), 2-rod levonorgestrel (LNG) implant (Jadelle), or Copper IUD. Participants completed a phone survey every 6 months over 24 months between December 2018 and March 2021. After the emergence of the COVID-19 pandemic, participants were re-contacted between September 2020 and April 2021 to complete an additional COVID-19 survey by phone about their experiences with SRH in the context of the pandemic.^1^ While participation in the original CUBE study required participants to still be using one of the ECHO contraceptive methods, due to switching and discontinuation during the CUBE study period, eligible participants in the COVID-19 survey could be using any or no method. We attempted to re-contact all study participants who had completed the 18-month CUBE survey: 390 in South Africa and 234 in Zambia.

In addition, a purposive subset of CUBE participants was contacted to complete qualitative IDIs regarding their contraceptive use during CUBE, as well as their experiences accessing SRH and contraceptive methods and services during the pandemic [25]. In line with the primary objectives of the original CUBE survey, which was to describe contraceptive use dynamics over time, the IDIs included people from each of four groups: switched to a different method between ECHO and CUBE, discontinued their ECHO method during CUBE, used their ECHO method throughout CUBE, and reported challenges getting implant or IUD removal. In addition, a group of key informants (KIs) were also contacted for IDIs in each country. A list of contraceptive providers, community advocates, and other local and national key stakeholders who were involved in family planning (FP) and SRH programs or service provision was purposively compiled, with representatives of public, private, and NGO sectors, and tertiary educational facilities. In some cases, there was only one individual in a key position, who was invited to participate (e.g. provincial/district managers responsible for family planning). Rural/district Ministry of Health representatives were recommended by provincial managers. Finally, members of community advisory boards were consulted and approached as community advocate representatives.

The original CUBE study protocol and additional COVID-19 module were approved by FHI 360’s Protection of Human Subjects Committee, the University of the Witwatersrand Human Research Ethics Committee (HREC), the University of North Carolina at Chapel Hill Institutional Review Board, and the University of Zambia Biomedical Research Ethics Committee (UNZABREC).

### Data collection and analysis

#### COVID-19 phone surveys

The COVID-19 survey focused on participants’ ability to access the contraceptive method(s) of their choice during the pandemic (including removal of implants and IUDs, as desired). Those results are currently under review [26]. While the survey included 67 questions, participants were asked a varying number applicable to their personal situation, depending on their reported difficulty accessing health services, if they had experienced a recent pregnancy, whether they were currently using a contraceptive method, and if not, if they had tried to obtain one. Asking participants about their contraceptive use in the context of COVID-19 presented an opportunity to also ask about interest in SRH services that could be used without needing to visit a health care provider. The present analysis focused on a module of 10 questions regarding participants’ interest in receiving instructions and materials to use different FP-related methods or services on their own without visiting a provider or community health worker in person. This language was used as a simple description of what is meant by “self-care”, since respondents might not have been familiar with that specific term. Each of the 10 FP-related methods or services was read individually and a short description was provided. The response options for interest in each method/service were on a 5-point scale ranging from 1— “Very interested” to 5— “Very disinterested” (see Additional file 1). Participant responses to the COVID survey were entered into a pre-programmed, password-protected and online data collection form in REDCap [29]. Research staff reviewed the data weekly, and participants were recontacted as needed to complete any missing or unclear responses.

#### Survey analysis

First, we linked each participant’s ECHO and CUBE data to her COVID-19 survey responses. We conducted a descriptive analysis of sociodemographic characteristics of respondents, using data from ECHO and CUBE, using chi-square tests to compare differences by country. To assess women’s interest in using the 10 different FP-related self-care methods or services, we conducted a descriptive analysis by country, providing summary proportions. We collapsed responses of interest in each of the methods or services into a binary variable with responses “Interested” (combining very interested and fairly interested) and “Disinterested” (combining neither interested/disinterested, fairly disinterested, and very disinterested).

#### In-depth interviews

Trained female interviewers conducted IDIs in-person, over the phone, or via a web-based platform such as Zoom, depending on participant’s preference, distance from the original ECHO study site, and social distancing/quarantine guidelines at the time of the interview. IDIs were conducted between August and November 2020 in South Africa and between November 2020 and June 2021 in Zambia. All IDIs were recorded and transcribed in English, or if conducted in a local language, translated and transcribed simultaneously into English using a transcription protocol [30].

The majority of IDI questions were related to access to contraception, but for one question, female study participants were read a short description of “self-care” and provided examples, including getting condoms and self-testing for pregnancy or HIV. They were asked if they had ever used any self-care services or commodities. Two of the questions asked to KIs in their IDIs were included in this analysis. KIs were also read a similar short description and asked if their facility counseled on self-care options and whether they believed that the use of self-care had changed because of the COVID-19 pandemic. In addition, KIs were told that there is some preliminary evidence that women may be able to remove their own IUDs and asked for their opinion regarding IUD self-removal. The IDI questions and additional probing text used in this analysis are available in Additional file 2.

#### IDI analysis

We used applied thematic analysis to analyze IDI data as part of our primary CUBE analysis [31]. A codebook was developed to structurally and thematically code the transcripts using NVivo 12 for each different research population (female participants and KIs) [32]. Through a process of coding 10% of transcripts, discussion of discrepancies, and codebook revision, three analysts achieved inter-coder reliability. All additional transcripts were coded, with frequent meetings to resolve discrepancies. A coding report on self-care was generated for each study population through NVivo 12 and analysts synthesized the data from the coding report by conducting inductive thematic analysis to identify major trends and thematic domains.

## Results

The final quantitative sample for the COVID-19 module included 537 female participants, 342 in South Africa and 195 in Zambia (an 86% response rate from the 18-month CUBE survey participants). A subset of 39 women participated in the IDIs, 20 in South Africa and 19 in Zambia. Thirty-six KIs participated in the in-depth interviews, 16 in South Africa and 20 in Zambia. Among them were 15 healthcare providers, nine Ministry of Health officials or Program Managers, and 12 community advocates. All IDIs were conducted in-person, with the exception of seven KI IDIs in South Africa.

Survey respondents in South Africa were significantly younger, had higher levels of education and lower mean parity, and were more likely to be students and not living with their current partner compared to those in Zambia. Consistent with the main CUBE sample [25], no significant differences were found in the contraceptive methods used at CUBE enrollment between countries. However, at the time of the COVID survey module, respondents in Zambia were significantly more likely to report no method use and less likely to be using copper IUDs, DMPA-IM, and condoms, compared to those in South Africa (Table 1).

**Table 1:** COVID-19 survey module participant characteristics, by country; n (%)

	South Africa (n = 342)	Zambia (n = 195)	Total (n = 537)	p-value
Age at COVID survey (mean, SD)^a^	27.8 (3.9)	29.6 (4.7)	28.4 (4.2)	< 0.001
19–24	88 (25.7)	48 (24.6)	136 (25.3)	< 0.001
25–30	195 (57.0)	73 (37.4)	268 (49.9)	
31–39	59 (17.3)	74 (38.0)	133 (24.8)	
Level of education^b^				< 0.001
No schooling	0 (0.0)	13 (6.7)	13 (2.4)	
Primary school	0 (0.0)	73 (37.4)	73 (13.6)	
Secondary school, not complete	108 (31.6)	77 (39.5)	185 (34.5)	
Secondary school, complete	148 (43.3)	25 (12.8)	173 (32.2)	
Attended post-secondary school	86 (25.2)	7 (3.6)	93 (17.3)	
Parity (mean, SD)^b^	1.2 (0.9)	2.6 (1.3)	1.7 (1.3)	< 0.001
Employment status^c*^				< 0.001
Homemaker/Unemployed/Other	183 (53.8)	134 (69.4)	317 (59.5)	
Student	61 (17.9)	4 (2.1)	65 (12.2)	
Part or full-time employment	96 (28.2)	55 (28.5)	151 (28.3)	
Partner status^c**^				< 0.001
Living together (married/unmarried)	20 (5.9)	172 (88.7)	192 (36.0)	
Not living together (married/unmarried)	303 (89.1)	21 (10.8)	324 (60.7)	
No current partner^***^	17 (5.0)	1 (0.5)	18 (3.4)	
Method at ECHO exit/CUBE enrollment				0.69
LNG implant (Jadelle)	113 (33.1)	70 (35.9)	183 (34.1)	
Copper IUD	89 (26.0)	45 (23.1)	134 (24.9)	
3-month injectable	140 (40.9)	80 (41.0)	220 (41.0)	
Contraceptive method using at COVID survey				< 0.001
None	34 (9.9)	61 (31.3)	95 (17.7)	
LNG implant (Jadelle)	77 (22.5)	47 (24.1)	124 (23.1)	
ENG implant (Implanon)	5 (1.5)	3 (1.5)	8 (1.5)	
Copper IUD	72 (21.1)	29 (14.9)	101 (18.8)	
2-month injectable	3 (0.9)	0 (0.0)	3 (0.6)	
3-month injectable	107 (31.3)	46 (23.6)	153 (28.5)	
Oral contraceptives	4 (1.2)	6 (3.1)	10 (1.9)	
Male/female condoms	39 (11.4)	2 (1.0)	41 (7.6)	
Standard Days Method/Cycle Beads	1 (0.3)	1 (0.5)	2 (0.4)	

^a^Calculated based on age at ECHO enrollment and date of COVID survey; ^b^Collected at ECHO enrollment; ^c^Collected at CUBE 24 month survey; *2 missing from South Africa and 2 missing from Zambia; **2 missing from South Africa and 1 missing from Zambia; ***Includes widowed/separated/divorced; LNG: levonorgestrel; IUD: intrauterine device; ENG: etonogestrel

Female IDI respondents were similar in overall demographics to the full survey sample, with respondents in Zambia slightly older with higher parity. However, more women interviewed in South Africa were currently not using an FP method than those interviewed in Zambia. KIs interviewed were overwhelmingly female and had mean ages in their 40s in both countries (Table 2).

**Table 2:** IDI participant characteristics, by country; n

Women	South Africa (n = 20)	Zambia (n = 19)
Age (mean (range))	27.7 (21–35)	29.3 (22–37)
Parity (mean (range))^a^	1.3 (0–3)	2.6 (1–5)
Partner status^b^		
Living together (married/unmarried)	1	18
Not living together (married/unmarried)	17	1
No current partner	2	0
Contraceptive method using at time of IDI		
None	5	1
LNG implant (Jadelle)	4	2
ENG implant (Implanon)	0	1
Copper IUD	2	5
2-month injectable	1	0
3-month injectable	3	7
Oral contraceptives	0	2
Male/female condoms	5	0
Standard Days Method/Cycle Beads	0	1

Key informants	South Africa (n = 16)	Zambia (n = 20)
Female	14	18
Age (years) (mean (range))	47.3 (26–66)	42.0 (28–68)
Position		
Healthcare provider	6	9
Ministry of Health officials/Program managers	6	3
Community advocate	4	8

^a^Collected at ECHO enrollment; ^b^Collected at CUBE 24-month survey; LNG: levonorgestrel, IUD: intrauterine device, ENG: etonogestrel

### Women’s interest in SRH self-care methods and services

**Fig. 1:**
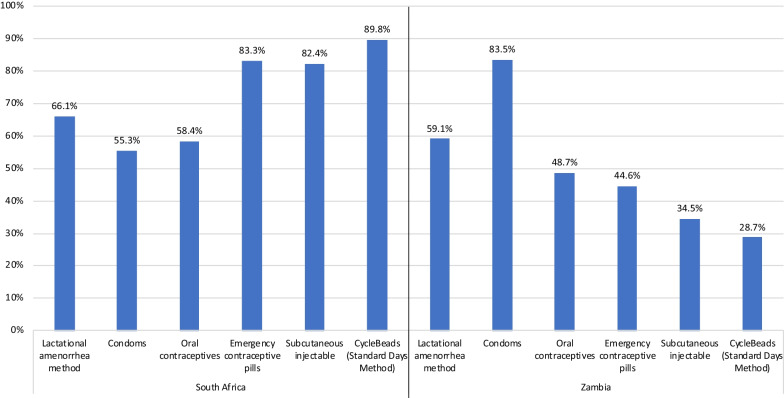
Survey participant reported interest in getting instructions and materials for specific self-care contraceptive methods in South Africa (N = 342) and Zambia (N = 195)

**Fig. 2:**
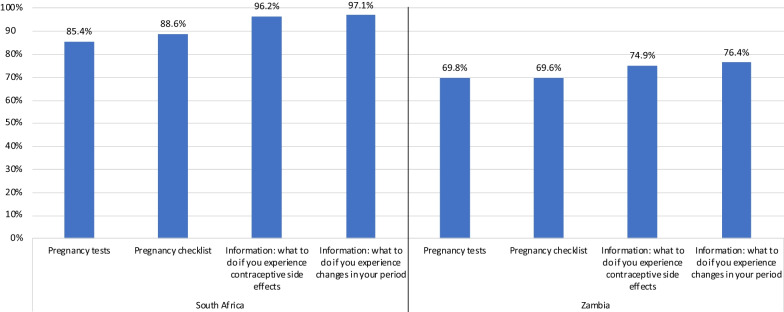
Survey participant reported interest in getting instructions and materials for FP-related self-care tests, tools, and information in South Africa (N = 342) and Zambia (N = 195)

Survey respondents in South Africa were more interested in getting instructions about and materials to use ECPs, subcutaneous injectables, and CycleBeads, while Zambian respondents were most likely to say that they wanted more information and access to condoms (Fig. 1). While still mentioned by approximately half of respondents, interest in condoms was the lowest of the methods mentioned in South Africa, followed by OCPs. In Zambia, only around a third of respondents were interested in subcutaneous injectables and CycleBeads. Most South African respondents were interested getting instructions and materials for pregnancy tests, pregnancy checklists, with almost all wanting information on managing contraceptive-related side effects or changes in menstruation (Fig. 2).

### Women’s use of SRH and contraceptive self-care methods and services

Of the 39 female IDI respondents, most reported limited use of self-care methods, with only a few women ever having used self-administered pregnancy tests (n = 4 in Zambia), HIV self-testing (n = 2 in Zambia), or ECPs (n = 2, one each in South Africa and Zambia). However, over a third of respondents in both countries had previously used male or female condoms. One respondent in South Africa discussed other traditional “self-care” remedies, such as taking over-the-counter antacids, aspirin, or antibacterial drugs to clean out the vagina and prevent pregnancy after sex, while ultimately acknowledging that these self-remedies do not always work. Overall, women’s IDI responses were very short, without much additional detail on their experiences with self-care methods.

### Key informant opinions of and recommendations for SRH and contraceptive self-care methods and services

#### Self-care methods or services counseled on or provided

Key informants spoke to the specific SRH self-care services that they counsel on and/or provide to women. A couple of KIs reported counseling women on home pregnancy testing, since knowing their pregnancy status could help in a woman’s decision making and next steps.

If a client came in…late for a re-injection…usually you give the Depo and counsel the patient that, you know, “you need to do a pregnancy test in a month's time” ... And if [*the*] pregnancy test is negative, then it’s all well because you’ve already been injected and you are safe. But if it’s positive, “do you want to keep the pregnancy or don’t you want to keep the pregnancy.” If you do, then you wait and you go to antenatal clinic and register at 6 weeks and then you carry on the pregnancy. If you don’t want to keep the pregnancy, as soon as you do a pregnancy test, it’s positive, come back, [*we’ll*] give you a letter for the termination of the pregnancy… [**Professional Nurse in South** **Africa]**

Three KIs in both countries described providing HIV self-testing, especially as a resource for reducing volume in the clinic, but another KI in Zambia was concerned that people take them home, but do not use or waste them.

Some people really used…the self-test kits. But for some of them, they care nothing. Why? Because they sometimes could get the kit, but don’t bring their results back. Meaning that they got the test and just dumped them. [**Community advocate in Zambia**]

Counseling on contraceptive self-care was not universal across methods. Four KIs from both countries noted that condoms are the main self-care method they discuss with clients, especially if other methods are out of stock. Similarly, four KIs mentioned counseling on or providing ECPs. One KI in South Africa expressed discomfort with people using ECPs as their primary method, while another (also in South Africa) stated that information on ECPs should be widely disseminated, especially during COVID. Counseling on subcutaneous DMPA (DMPA-SC) for self-injection seemed to be related to its availability. Two Zambian KIs described providing DMPA-SC, while other KIs in both countries reported not having access to the product. One KI in Zambia noted how important the availability of self-care methods like DMPA-SC is in ensuring continued method use:

During the COVID-19 pandemic, we didn’t have methods...like the Sayana Press [*brand name of DMPA-SC*], which we can give to the women to be injecting to themselves at their homes, we never had during that period [*the COVID-19 pandemic*]. But the counseling was being done to them in case we have them so that they know how to inject themselves at home. [**Midwife in Zambia**]

Nine KIs, mostly in Zambia, emphasized that COVID-19 led to an increase in SRH self-care counseling, contraceptive method provision, and provision of other self-care resources. This included increased use of DMPA-SC, HIV self-testing, and provision of condoms and additional months of OCs to reduce the need to return to the facility for re-supply. One KI in Zambia also mentioned counseling clients that condoms and OCs can be obtained from private pharmacies if people do not want to come to the facilities because of COVID.

Self-care for some mothers that surely do not want to come to the facility, they come to seek guidance from us…We’ve advised a lot on the use of condoms for those that don’t want to come to the facility. They are afraid, they even say to us “we are afraid to come, we thinking maybe we can have this corona”. So we’ve advised them to use condoms if they are able to… or rather maybe, they can just purchase this Microgynon, Microlut, [*brand names of OCs*] from the pharmacies. [**Registered nurse in Zambia**]

#### Reasons for NOT counseling on or providing self-care methods or services

One KI in South Africa and two in Zambia reported that they do not counsel on or provide methods for self-care, because they are concerned that women may harm themselves and/or blame the provider for any difficulties they experience.

Usually we don’t advise any clients to go for self-care. We always tell them either to come here or go to the nearest facility around them instead of going for the over-the- counter self-care, because they might end up doing the wrong thing, which at the end of it all, it will fall back on us. [**Registered Nurse in Zambia**]

Another South African KI mentioned that they do not think people are necessarily that interested in self-care since there are so many locations to access health services.

#### Potential for women to self-remove IUDs

An area of inquiry that received a lot of feedback was the potential for IUD self-removal. After being introduced to the concept and preliminary evidence regarding IUD self-removal, only two KIs (one from each country) thought that women could possibly self-remove their IUDs if provided education, saying it would provide greater reproductive autonomy for some women:

We have not educated them on self-removal of IUDs, but I have an opinion to say if they can be educated, maybe do the demonstration…They can do it for themselves...If they are empowered, yes. They can do it…. That would give her a chance to say, “Okay, they have inserted it, but when I want to remove it, I will be able to remove it”. [**Rural Clinic Manager in South Africa**]

The majority of KIs in both countries felt that women should return to a health facility to have their IUDs removed and gave both provider-focused and women-focused reasons. For example, a couple of KIs in South Africa expressed how women coming back to the facility for IUD removal provides the opportunity for the provider to offer additional services:

For one, I think it’s a good opportunity to look at the cervix at that time and also the number of reasons why she’s removing it, if she’s removing it because she wants another pregnancy…I mean if she’s due for a Pap smear that’ll be an opportune moment to actually do a Pap smear for her, you could treat an STI if she had one…[**Professional** **Nurse in South Africa**]

Nine KIs (more in Zambia than South Africa) expressed concern that women might introduce infections or injure themselves by attempting or performing self-removal.

I think it’s not safe.... because with us, at the facility, you are able to examine the woman, and see if it is infected. So if they do it at home, they won’t even be able to see that there’s an infection or something. So maybe they can just even pull it out. Ya, which is not safe, and they may injure themselves and may traumatize themselves. And then here we use sterile equipment...when removing that. [**Midwife in Zambia**]

Other concerns brought up by KIs were related to women experiencing difficulty with self-removal, the length of the strings needed for self-removal (e.g., whether partners will be able to feel them), issues with partial removal, and partner interference with removal, as has been seen with some implant users.

A couple of KIs in South Africa questioned why self-removal of IUDs is needed, stating that they think people can wait or make a plan to get to a clinic if they really want removal. However, two KIs in South Africa acknowledged that even though they do not think self-removal is a good option in general, they recognized that getting removal in a private facility can be costly and that self-removal might be an option under COVID-19 conditions, possibly in consultation with a healthcare provider.

So I don’t think it’s a good idea, but, in times of a pandemic, which, you know, doesn’t happen every day… if the women is able to contact you telephonically… and discuss with you… it is an option, but I don’t think it should be routinely provided. [**OB/GYN in South Africa**]

#### Barriers to accessing self-care methods

KIs mentioned many barriers to accessing SRH and FP self-care methods or services, especially in South Africa. Respondents noted that access to ECPs and HIV and pregnancy tests were particularly limited due to cost of transport and lack of pharmacies in townships or rural areas, and these were compounded by COVID movement restrictions:

Emergency contraceptives under [*COVID-19*] lockdown [*level*^2^] 4 or 5, even 3, was actually difficult. Because access to town was a problem. To come, and you can imagine, there are no pharmacies in the townships, most of them...Patients would come asking for emergency contraceptives 96 hours later. And you tell them, “look, you’re late”… [**OB/GYN in South Africa**]

KIs also noted that while self-care methods might be more available in the private sector, that meant a cost that not everyone could afford.

Five KIs, mostly in Zambia, reported that they were concerned that women’s general lack of reproductive knowledge would limit their ability to use self-care methods or to use them correctly and other KIs reiterated that women did not understand what was meant by “self-care”:

Okay, you’ve mentioned a lot of things that fall under self-care. So looking at the community that we are catering for...it’s not a very, for lack of a better term, “learned community,” so we really don’t advise for them to start doing self-care because they might do it the very wrong way, and then they’ll come and blame us. [**Certified midwife in Zambia**]

Finally, one KI in South Africa mentioned that it may be better for some stigmatized services and populations (e.g., HIV self-testing for people who sell sex) to seek care at a facility, rather than self-care at-home or through mobile outreach where other people might see what they are using and ask questions.

#### Recommendations for expanding self-care in the future

##### For disseminating information

KIs also provided recommendations for ways to disseminate information on self-care. One KI in South Africa mentioned radio spots that have been deployed regarding HIV self-testing as part of a national strategy and another mentioned social media (especially for youth), plus in-person counseling sessions with physical demonstration of the methods:

The best strength that you can actually teach them is when you…gather in one place and then you speak to them, and then you send the information. That’s sometimes more effective than we’ve seen because when we give even digital stuff without actually having a physical interaction with them, they don’t actually learn that way. But they learn more effectively when you see them, and they get to see the methods. So... when you speak about any...contraception they see them, they touch them, then they understand, “oh, that’s what it is”. [**Community Advocate in South Africa**]

Two KIs in Zambia suggested easy-to-follow, non-technical, possibly pictorial instructions for women to refer to if they forget how to use a method (particularly for DMPA-SC).

The kind of IEC [*information, education, and communication*] materials that could be used should be something that is simple to understand with the community because, if we put into technical language, not everyone knows how to read...It can be not only a cartoon, but in a way of messaging or breaking down into a cartoon or into different languages that they could read and relate. [**Community Advocate in Zambia**]

##### Making self-care methods more widely available

Finally, KIs shared their recommendations for making self-care methods more widely available. Suggestions included making all contraceptive methods more available on an outpatient basis and making people aware that self-care options are available and where they are available.

Okay, in order for there be a change or increase or anything like that…the youth has to be first aware that there are self-care services or management that are available out there…Like, the condoms, the only thing that they know about is where they can get [*them*] in the bathrooms or in health facilities, but all the other ones that you’ve mentioned, the calendars check, the pregnancy test, those things are not available. I don’t even know, even as you’re talking to me now, I’m wondering…where are those packs available? [**Community Advocate in South Africa**]

## Discussion

In this study we found that few women in South Africa and Zambia have used SRH methods or services defined as “self-care” by WHO (apart from condoms), even in the context of the COVID-19 pandemic. However, South African respondents were interested in learning more, especially about ECPs, subcutaneous injectables, and CycleBeads, while Zambian respondents wanted more information and access to condoms. Key informants, including healthcare providers, generally endorsed benefits of SRH self-care, but some, especially in Zambia, had reservations about women’s ability to utilize these methods and services. Our findings highlight that even though women are interested in self-care methods, mixed views of providers and other KIs could prevent wider dissemination of self-care options.

While survey respondents expressed interest in SRH self-care, most IDI respondents in this study had little direct experience with it. In addition, the COVID-19 pandemic exacerbated lack of access to self-care options such HIV and pregnancy testing because of movement restrictions [26] and increased stock-outs of contraception, especially injectables in South Africa [33]. This context underscores that self-care can be particularly important for reducing contraception discontinuation and improving access to other SRH services. For example, registration of self-administered injectable contraception and introduction into the health system in South Africa and increased provision in both countries could reduce the need to return to facilities for injections, and making HIV self-testing kits more widely available could facilitate increased access to this service [34] in light of future shocks to the health system.

One way to potentially improve access to SRH self-care methods and services that female respondents expressed higher interest in, such as ECPs, condoms, and pregnancy tests, would be to make them available through the private sector. However, the costs of contraceptive methods and other SRH self-testing supplies, as well as physical accessibility of pharmacies and drug shops may still limit access to only those with resources. For example, as noted by key stakeholders in South Africa, costs are higher in the private sector, fewer pharmacies exist in rural or under-resourced areas (like townships), and, during the COVID-19 pandemic, extended periods of restriction on movement limited access outside immediate areas of residence. Therefore, introduction of self-care methods, such as over-the-counter OCs, into pharmacies, without considering other policy changes or innovations to service delivery will likely not be sufficient. Examples of policy changes from other settings that might increase access include advance provision of ECPs or pregnancy tests at health facilities and providing multiple months of OCs and doses of self-administered injectables during a single clinic visit. While this study did not ask respondents about their preferred source of self-care methods, it is an important topic for future inquiry to ensure access to these methods and supplies in convenient and discreet locations, whether that is the public or private sector or both.

While expanding access to SRH self-care is crucial, it is important to underscore that not all people want to use SRH methods and tests themselves and some prefer to have some level of interaction with a healthcare provider. For example, people who desire covert contraceptive use, possibly due to partner opposition or fears of gender-based violence, may want to obtain their method at a facility [35]. Also, as noted by one KI in this study, specific populations, such as people who sell sex, may not prefer SRH self-care for privacy and stigma-related reasons. Other factors limiting interest in and uptake of self-care methods cited in previous research include difficulty understanding where to access or how to use self-care products due to low education or illiteracy, fear of complications due to incorrect use, and concerns about potential lack of support if complications occur [20]. In line with its stated principles, self-care should be person-centered and responsive to the life circumstances and preferences of individuals [1]. While this approach recognizes the importance of encouraging people’s active participation as advocates for their own healthcare, such participation may very well include the involvement of a health care provider.

As mentioned above, providers and key stakeholders had mixed opinions about the role of SRH self-care. More providers in South Africa reported seeing the benefits of educating women on these options and making them more available throughout the health system, while more Zambian providers were worried about women’s education levels and their ability to use the methods “correctly” and not “misuse” them, a concern found in prior research of providers’ perspectives on SRH self-care [2, 20]. Similarly, providers asked about self-injectable contraceptives in Nigeria and Uganda reported biases regarding who they believed was not suitable to use the method, such as those with less education or income [36]. Such attitudes can also influence providers’ choices to stock certain methods, limiting their use. While one potential benefit of increased use of SRH self-care is reduced burden on providers’ time and costs to health systems, future trainings will need to balance providers’ beliefs about their own expertise, positionality, and perceived responsibility for patients’ health with the potential benefits of supporting those people who want to be more active participants in their own health care. Development of specific national guidelines outlining key principles for self-care could encourage providers to reconsider their beliefs and practices. For example, the national self-care guidelines in Uganda propose an approach with graduated assistance for young adolescents and those who are unable to read or understand instructions to receive initial assisted self-care until they can take on this responsibility independently [37]. Nigeria’s self-care guidelines propose a Basic Health Care Provision Fund that includes self-care intervention products, so they are accessible to all people and not just those with resources [37].

Most KIs interviewed were not supportive of the idea of IUD self-removal, though a small number mentioned that they think that women can be educated on how to do it and some acknowledged that it might be an option during emergencies like COVID-19. In both countries, IUD use is very low (< 2% of contraceptive users) [3, 4] and the low prevalence of trained healthcare providers may contribute to a lack of familiarity with IUDs and a reluctance to endorse self-removal. One current barrier to IUD use is accessing removal from a health facility. A recent study in Senegal found that 55% of IUD users reported challenges getting removal, with the most common reasons being long wait times, difficulty getting away from the house or finding money to pay for transport or services, or the provider was not available [38]. Limited research from the US in 2014 has shown that, when educated on how to do so, about 20% of women willing to try IUD self-removal were able to remove [18], and more recent analyses of online forums show that there are women interested in this option, most often due to costs and lack of appointment availability [39]. Online forums and videos provide tips on removal and show successful attempts, underscoring how some women feel confident about taking this aspect of their reproductive health into their own hands [40, 41]. Our research on this topic is some of the first to assess opinions regarding IUD self-removal in sub-Saharan Africa. While efforts to make hormonal IUDs more widely available are accelerating [42], myths and misconceptions about IUDs persist, including that it may migrate in the body [43]. More research on women’s interest in self-removal and formal studies of their ability to remove are needed in this context, especially if the potential for self-removal makes the method more attractive to some users [19].

This study had limitations. Most importantly, this study was conducted from August 2020 to June 2021, when the COVID-19 pandemic was an ongoing worldwide public health emergency. Therefore, it is possible that female respondents and KIs expressed more interest or support for self-care than they would have in standard health care conditions. However, as we documented in the results of the COVID-19 survey, most respondents did not report difficulty accessing their preferred contraceptive method at the time [26]. This provides some initial evidence that there is general interest in these methods and services, not just in emergency situations. While understanding the use of and interest in self-care methods and services for SRH was a component of the main study, it was not the main research question and, as such, the topics were not explored or probed in an in-depth manner, especially in the IDIs with female participants. Perhaps as a result, the qualitative data on self-care from women were limited. In addition, participants were not explicitly asked if they would definitely want to use the self-care methods and tools, but were asked if they would like to get instructions and materials. It is possible that participants who were already familiar with one of the methods or tools described in the quantitative survey stated that they were not interested in learning more, potentially underestimating the prevalence of people who have interest and are already actively using one or more of the methods. However, these estimates still provide some baseline information about the volume of interest for more information, which may be particularly useful for tests or methods that are newer to the marketplace in that country or tools that have been historically underutilized. Moreover, study participants are not representative of all women of reproductive age in South Africa and Zambia, as they were previously enrolled in the ECHO study, so study findings may not be generalizable to the broader population. Finally, seven of the 16 KI IDIs in South Africa were conducted by phone, rather than in-person, which may also have impacted the extent to which the interviewer was able to observe a respondent’s facial and body cues, and may have contributed to less probing on certain topics. Further limitations have been described previously [25]. Despite these limitations, this study contributes to the growing knowledge base around opinions of and demand for SRH self-care among women, healthcare providers, and other key informants.

## Conclusion

This study found that few women in South Africa and Zambia have used self-care methods for SRH, but are interested in learning more about them. KIs, including health care providers, reported more mixed opinions, though they reported more frequently discussing self-care during the COVID-19 pandemic. Future research should confirm women’s desire for access to specific self-care methods as part of standard services outside of the unique conditions of COVID-19, as well as identifying populations that have particularly high demand. Healthcare provider perspectives are also essential, since without their acceptance and participation, access to self-care methods will remain stunted regardless of demand from users. Ensuring that access to self-care information, methods, and services is more than just a temporary measure deployed by health systems has the potential to increase access to SRH care and support women’s autonomy and healthcare needs.

## Supplementary Information

**Additional file 1.** Self-care questions asked in survey

**Additional file 2.** Self-care prompts used during IDIs

## Data Availability

The CUBE COVID module dataset supporting the conclusions of this article is available from Harvard Dataverse (10.7910/DVN/TJKRU4). The qualitative data generated and analyzed during the current study are not publicly available as full transcripts for ethical reasons because even after removing directly identifiable information such as names, participant identify may be difficult to fully conceal and research locations may remain potentially identifiable, presenting a risk of deductive disclosure. However, relevant excerpts are available from the corresponding author Alice Cartwright (acartwright@fhi360.org), author Rebecca Callahan (rcallahan@fhi360.org) or institutional access via opendata@fhi360.org on request. ECHO data is available for researchers who provide a methodologically sound proposal, which will be reviewed by the ECHO Management Committee. Proposals should be directed to icrc@uw.edu; to gain access, data requestors will need to sign a data access agreement and any proposal will require approval by the ECHO Management Committee.

## Abbreviations

CUBE: Contraceptive Use Beyond ECHO
DMPA-IM: Intramuscular depot medroxyprogesterone acetate
DMPA-SC: Subcutaneous DMPA
ECHO: Evidence for Contraceptive Options and HIV Outcomes
ECP: Emergency contraceptive pill
FP: Family planning
IDI: In-depth interview
IUD: Intrauterine device
KI: Key informant
LNG: Levonorgestrel
OC: Oral contraceptives
SRH: Sexual and reproductive health
STI: Sexually transmitted infection
United States: US
WHO: World Health Organization

## Footnotes

1 The government of Zambia enacted a series of public health measures related to COVID-19 on March 13, 2020 and the government of South Africa declared a National State of Disaster on March 15, 2020 [27, 28].

2 In South Africa, there were five lockdown levels, with level five the most restrictive and one the least. Level five included the most extreme restrictions to contain the spread of COVID-19: all businesses were closed except those providing essential services, public transport was limited, and travel between provinces was not permitted (with the exception of funerals). Level four allowed some activity to resume, but was mostly limited to agricultural, mining, and professional services. Health clinics were open during lockdown, but experienced staff shortages and prioritization of COVID-19.
