# Supplementary material for: Perspectives on sexual and reproductive health self-care among women, healthcare providers, and other key informants: a mixed-methods study in South Africa and Zambia
Source: Reprod Health. 2023 Apr 28;20:65. doi: 10.1186/s12978-023-01596-x (PMC10144905; doi:10.1186/s12978-023-01596-x)
Supplement: Supplementary file 1 — Additional file 1. Self-care questions asked in survey [file 12978_2023_1596_MOESM1_ESM.docx]

**Additional File 1: Self-care questions asked in survey**

“We would like to understand what family planning services women like you may be interested in using on their own without seeing a provider or a community health worker in person. For each of the following, how interested would you be to get instructions and materials to use it yourself?”

|  | Very interested | Fairly interested | Neither interested nor disinterested | Fairly disinterested | Very disinterested |
| --- | --- | --- | --- | --- | --- |
| 1. Pregnancy test (Women can purchase a urine test to determine pregnancy from a pharmacy, drug shop, or potentially online depending on the country.) |  |  |  |  |  |
| 1. Pregnancy checklist (A series of questions to assess whether you are pregnant without taking a pregnancy test.) |  |  |  |  |  |
| 1. Lactational amenorrhea method or LAM (Women who just had a baby and meet three criteria are protected from pregnancy. Women must be exclusively breastfeeding, have not resumed menstruating, and are 6 months or less postpartum.) |  |  |  |  |  |
| 1. Condoms |  |  |  |  |  |
| 1. Pill (Women can take a pill every day to avoid becoming pregnant. Pills may be purchased at a pharmacy, drug shop, or online depending on the country.) |  |  |  |  |  |
| 1. Emergency contraception (As an emergency measure after unprotected sexual intercourse women can take special pills at any time within three to five days to prevent pregnancy. Pills may be purchased at a pharmacy, drug shop, or online depending on the country.) |  |  |  |  |  |
| 1. Subcutaneous injectable (A contraceptive injection administered via a small needle that you could give yourself after a provider or CHW shows you how to do it. This is a much smaller needle than ones you may be familiar with that are used for other types of injectable contraceptives.) |  |  |  |  |  |
| 1. Standard days method (Cycle Beads) (A woman can use a string of colored beads to know the days she can get pregnant. On the days she can get pregnant, she and her partner use a condom or do not have sexual intercourse.) |  |  |  |  |  |
| 1. Sometimes women experience side effects like changes in weight, mild nausea, headaches, or fatigue when they use a family planning method. How interested would you be in receiving information on what to do if you experience side effects without seeing a provider or CHW? |  |  |  |  |  |
| 1. Sometimes women experience changes in their period when they use a family planning method. How interested would you be in receiving information on what to do if you experience changes in your period without seeing a provider or a CHW? |  |  |  |  |  |
