# Supplementary material for: Perspectives on sexual and reproductive health self-care among women, healthcare providers, and other key informants: a mixed-methods study in South Africa and Zambia
Source: Reprod Health. 2023 Apr 28;20:65. doi: 10.1186/s12978-023-01596-x (PMC10144905; doi:10.1186/s12978-023-01596-x)
Supplement: Supplementary file 2 — Additional file 2. Self-care prompts used during IDIs [file 12978_2023_1596_MOESM2_ESM.docx]

**Additional File 2: Self-care prompts used during IDIs**

**To women:**

“’Self-care’ includes health practices that you can use yourself without needing to access them from a health provider.  Have you made use of any self-care options related to family planning during the pandemic?

*Probe for HIV self-testing, pregnancy testing, contraceptive options (including fertility awareness methods, male and female condoms, EC, birth control pills).*

**To key informants:**

“Self-care interventions are a way for individuals to obtain products or diagnostics fully or partially separate from formal health services that can be used with or without the direct supervision of a health worker. Examples of self-care interventions include self-injectable contraception (e.g., DMPA-SC), fertility awareness methods or HIV self-tests, pregnancy self-tests, male and female condoms, emergency contraception, and over the counter OCs. How has your facility been counseling on self-care during the COVID-19 pandemic, if at all?”

“There is some evidence that when properly counselled, some women are able to remove their own IUDs. What are your thoughts about IUD self-removal for women who want to stop using their IUD and are unable to get to a facility providing removal services?”
